# Perinatal Outcomes in Babies Born before Arrival at Prince Mshiyeni Memorial Hospital in Durban, South Africa

**DOI:** 10.1155/2022/2316490

**Published:** 2022-02-05

**Authors:** M. Jenneker, N. R. Maharaj

**Affiliations:** ^1^Department of Obstetrics and Gynaecology, School of Clinical Medicine, College of Health Sciences, University of KwaZulu-Natal, Durban, South Africa; ^2^Department of Obstetrics and Gynaecology, Prince Mshiyeni Memorial Hospital, Umlazi, Durban, South Africa

## Abstract

**Objective:**

To evaluate the maternal demographics, incidence, perinatal outcomes, and characteristics of babies born before arrival (BBAs) to hospitals.

**Methods:**

A prospective, observational study was conducted at a large maternity unit in Durban, KwaZulu-Natal. A total of 200 mothers who attended the hospital within 24 hours of an out-of-hospital birth were recruited and interviewed, and 142 participants were eligible. A total of 128 mothers who delivered their babies in hospital (inborns) were used as the control group. Specific maternal and neonatal characteristics were analysed.

**Results:**

The incidence of BBAs was 2.2%. The percentage of premature neonates in the BBA group was 54% vs 17.9% for inborns (*p* ≤ 0.001). A total of 33.8% of BBA mothers were unbooked vs 2.4% of inborns (*p* ≤ 0.001). The majority (59%) of inborns were primigravidas whereas the majority (73.9%) in the BBA group were multigravidas (*p* ≤ 0.001). Women in the BBA group were more prone to genital tears (*p* ≤ 0.001). There were no significant differences in respect of NICU admission and all-cause mortality; however, an increased risk for hypothermia and hypoglycaemia was found.

**Conclusion:**

BBAs are at a significant risk of prematurity, low birth weight, hypothermia, and hypoglycaemia and are prone to longer hospital stays.

## 1. Introduction

In South Africa (SA), the majority of babies are born in hospitals under controlled settings [1]. An unknown proportion of babies who are born outside of the hospital are planned home births in the presence of trained birth attendants [1]. However, several babies are born outside of the hospital in the absence of trained personnel [2–4]. These babies are referred to as babies born before arrival (BBAs) to hospital.

There is a paucity of data in developing countries on the incidence of BBAs. In 2009, an incidence rate of 5.7% in KwaZulu-Natal (KZN) and 5% in Gauteng was reported by the National Department of Health [1]. Furthermore, prevalence rates vary globally, and prevalence rates in South Africa are not well established. Evidence suggests that BBAs represent a vulnerable and high-risk population group who are susceptible to poor perinatal outcomes [2–4]. They are associated with increased complications such as low birth weight and prematurity [1]. In SA, the primary obstetric reasons contributing to perinatal mortality are prematurity and birth asphyxia [5, 6]. The identification of susceptible groups of women and implementation of relevant interventions are therefore necessary to reduce poor perinatal outcomes.

The World Health Organization (WHO) has stated that countries should aim for a reduction in the under-five mortality rate (U5MR) and neonatal mortality rate (NMR) to meet the sustainable developmental goal three (SDG 3) “good health and wellbeing” by 2030 [7]. This can be described as a goal within a goal; of the thirteen targets, nine are outcome targets with target 3.2, i.e., reducing all preventable deaths under five to as low as 25/1000 and reducing neonatal mortality rate to as low as 12/1000 live births. Globally, the U5MR was reduced by 43% since 2000, with recorded deaths placed at 43 deaths per 1000 live births and the NMR reduced from 31/1000 to 19/1000 in 2015. The WHO recommendation is that although the NMR has improved, the proportion of deaths under five is increasing, indicating an urgent need to improve antenatal and perinatal care.

Various studies have analysed NMRs and interventions to prevent poor outcomes in BBAs [6]. BBAs contribute to poor perinatal outcome and higher neonatal mortality rates [7]. Lack of transport from home, reduced antenatal attendance, poor immediate care of the newborn, and low birthweight babies in particular are noted as important risk factors for asphyxia, morbidity, and mortality [8]. It has been reported that babies born before arrival may have a twofold increase in morbidity and an 11-fold increase in mortality when compared to babies born in hospital [9]. This study also found that despite the relatively low incidence of BBAs (2%), they contributed 17% to the neonatal mortality rate [9].

With this background, we conducted a study to evaluate perinatal outcomes in BBAs who presented at a large maternity unit in Durban, SA. The primary objective for the study was the comparative assessment of perinatal morbidity and mortality in BBAs and babies born in hospital. In addition, the study sought to evaluate the incidence of BBAs and identify the characteristics of mothers and their babies. The purpose of the study was to improve our understanding of BBAs by identifying risk factors, evaluating outcomes, and developing recommendations to improve perinatal care in this group of patients.

## 2. Materials and Methods

### 2.1. Study Setting

The study is a prospective, observational case-control study and was conducted at the maternity unit at Prince Mshiyeni Memorial Hospital (PMMH). This is a large regional hospital in Durban, SA, that serves a predominantly Black African semiurban population.

### 2.2. Study Participants

The study included all mothers who delivered their babies outside the hospital labour ward. A delivery in the hospital car park, ambulance, or in the admission room was included in the study. The sample size included 142 cases and 128 controls. The time interval for presentation of a BBA to the hospital was 24 hours, in accordance with the eligibility criteria for enrolment. BBAs may have been delivered by a trained or untrained birth attendant, and the minimal accepted birth weight for enrolment was 500 g. All stillbirths were included in the study, and all cases were chosen irrespective of the maternal age. The control group comprised of women who presented to the labour ward or delivery suite. Maternal deaths, mothers with severe maternal morbidity, and intensive care unit (ICU) admissions were excluded from the study as per the eligibility criteria for enrolment. This was related to the concept of signing informed consent prior to enrolment which would have been compromised in severely ill or moribund patients. However, the study did not report any ICU admissions or maternal deaths. Grief counselling was offered to all mothers with stillbirths or poor perinatal outcome in accordance with the standard care of practice at the institution.

A systematic sampling strategy was employed in the study. All BBAs were compared with the first consecutive baby born in the hospital. The women who presented with a BBA were screened and if eligible, were consented prior to enrolment into the study. The informed consent document was administered prospectively to eligible participants. A standard operating procedure detailing the entire interview and a visit checklist were created to facilitate enrolment.

Inborns were sampled by a retrospective chart review using the labour ward birth register. Morbidity included prematurity, congenital abnormalities, neonatal sepsis, birth asphyxia, neonatal intensive care unit (NICU) admission, hypothermia, and hypoglycaemia. The neonatal characteristics included gender, birth weight, gestational age, and apgar score. Apgar scores were not assessed for BBAs instead mothers had to report if their babies cried or not at birth. The birth weight categories utilised in the study were extreme low birth weight (ELBW) 500 g to 999 g, very low birth weight (VLBW) 1000 g to 1499 g, low birth weight (LBW) 1500 g to 2499 g, normal birth weight (NBW) 2500 g to 3999 g, and large birth weight more than 4000 g. The maternal profile included age, marital status, household income, employment, parity, antenatal care, rhesus result, HIV status, syphilis status, anaemia, history of smoking, and alcohol or drug abuse. Maternal outcomes included postpartum haemorrhage (PPH), genital tears, hypertension, retained placenta, and puerperal sepsis. The length of hospital stay for both groups was also assessed.

The study was approved by the Local Ethics Committee and Biomedical Research Ethics Committee, University of KwaZulu-Natal, BE 561/16. Permission was also obtained from the institution and the provincial Department of Health.

### 2.3. Statistical Analysis

Descriptive statistics consisting of summary statistics (range) for numerical data and frequencies for categorical data were used. Comparison between the groups was conducted using Pearson's chi-squared test and Fishers exact test for associations. The data was analysed using PSPP and Stata. A *p* value of <0.05 was considered statistically significant.

## 3. Results

Of the 200 women that delivered a BBA, 142 were eligible for enrolment and a further 128 women were enrolled in the comparative group. The total sample size was 270 women and 270 babies. The incidence of BBAs for the study was 2.2%. The maternal characteristics for both groups are highlighted in Table 1. In the BBA study population, 79% of women admitted that they had no funds to come to the hospital. Ninety-one percent of women enrolled in the study were unemployed, and the incidence of single mothers was 92.2%.

Table 2 shows the characteristics of the babies born. Perinatal and maternal outcomes are represented in Tables 3 and 4, respectively. Statistically significant associations were noted for booking status, parity, birth weight, prematurity, length of hospital stay, hypoglycaemia, hypothermia, and genital tears. The association with maternal anaemia and low birth weight and prematurity was significant. The association between HIV and prematurity was not significant. The association for PPH and the apgar score may be limited and biased since it was dependent mainly on the participant's verbal report rather than an objective assessment.

## 4. Discussion

The BBA rate of a country is considered an index for accessibility of perinatal care, and a value above 1.5% is generally regarded as unacceptable [10]. In our study, the incidence of BBAs was found to be lower than the overall incidence for SA (2.2% vs 5%). Parag et al. (2014) [1] found an incidence of 1.8% in a local retrospective study, comparable to rates in developed countries. The lower incidence in our study may be attributed to the semiurban study setting, with more efficient public transport, access to roads, and the accessibility to a large regional hospital in the area. The area also boasts 3 midwife obstetric units (MOU) including an in-hospital MOU for low-risk mothers.

Potential predictors related to socioeconomic circumstances were demonstrated across the BBA group viz. a young, single, unemployed female of poor socioeconomic status. A social history relating to alcohol or smoking showed that 12.7% of mothers in the BBA group and only 1.6% of mothers in the control group consumed alcohol or were smokers. Women that smoke and consume alcohol in pregnancy generally have poor antenatal attendance, are at risk of prematurity, and may therefore be at increased risk of an out-of-hospital birth [3].

The study further demonstrated that the multigravida are at higher risk (*p* ≤ 0.001) for BBA than primigravidae. This stands to reason as the multigravida may be at risk of more precipitous labour than their primigravid counterparts. This is consistent with studies conducted by Potter et al. (1984) [10], Khupakonke et al. (2017) [11], and Bassingwaighte and Ballot (2013) [12] who showed that multiparty, including a shortened second stage of labour, low maternal age, low education level, and poor access to transport, are associated with BBA [10–12]. The majority of mothers in the BBA group were also unbooked. Similar findings for the association between antenatal attendance and parity were found in a retrospective study conducted in 2010 [1]. The proportion of HIV-positive participants in the study population was 44.4% (Table 1). No significant associations were made between HIV and BBA, and HIV and low birth weight or maternal anaemia. This is contrary to a meta-analysis of a cohort study conducted by Xiou et al. (2015) on the association of HIV and low birth weight and prematurity [13].

Our findings on the positive association between anaemia and prematurity and low birth weight are consistent with findings from a systematic review conducted by Rahmati et al. (2017) which showed a significant association between low birth weight and maternal anaemia [14]. Maternal anaemia may be used as a preventative factor for low birth weight [14].

Our study also demonstrated that BBAs were associated with perinatal morbidity but not all-cause mortality. The most significant association was prematurity, as 54% of the BBAs were premature compared with 17.9% of inborn babies. A limitation of the study is defining the degree of prematurity. In view of the large unbooked status in the BBA population and therefore the absence of an early ultrasound scan for dating, gestational age was calculated using the last normal menstrual period and the paediatric Ballard score. Therefore, babies of uncertain gestation were defined as premature, <37 weeks based on these principles. Furthermore, using the birth weight to predict the gestational age has its own limitation as LBW babies may be constitutionally small or growth-restricted and not necessarily premature. Prematurity is an established cause of perinatal morbidity and mortality especially in developing countries [5, 6]; hence, reducing the incidence of BBAs may reduce the rates of prematurity and associated perinatal morbidity and mortality. Furthermore, premature babies are generally low birth weight and hence require longer hospital stays for the mother and the baby. The statistically significant relationship with length of hospital stay in our study may be attributed to kangaroo mother care, intended to allow for a more reasonable birth weight before discharge.

Despite the high prematurity rate, most of the neonates had satisfactory outcomes. This was attributed to timeous neonatal resuscitation and optimal nursery care. Other contributing factors to improved outcomes included earlier presentation of mothers to the hospital. The average time to hospital was categorised as <1 hour, 1–5 hours, and >5 hours. The majority of women indicated that the average time to hospital was between 1 and 5 hours. Moreover, all mothers wrapped their babies in a blanket immediately after birth.

The majority of the women in the BBA group did not provide skin to skin (70%) nor did they breastfeed their babies (72%). These are important early measures for the prevention of neonatal hypoglycaemia and hypothermia, respectively. Bateman et al. (1994) showed that significant reductions in neonatal morbidity can be achieved with these simple interventions [3]. Moreover, the more significant finding of hypothermia and hypoglycaemia in the BBA group may be confounded by the higher proportion of premature and LBW babies in this group. Satisfactory outcomes in hypothermic and hypoglycaemic babies were attributable to resuscitation measures by the in-house paediatricians immediately on arrival to hospital (Table 2).

In terms of maternal outcomes, there was a statistically significant association for 1^st^ and 2^nd^ degree tears amongst BBA mothers. This may be attributed to a lack of supervision or assistance during childbirth; however, all mothers received immediate care with appropriate suturing of the area and had satisfactory outcomes.

### 4.1. Limitations

The diagnosis of postpartum haemorrhage (PPH) was based on patient enquiry and is limited by the absence of quantification of blood loss by a health care worker; however, there was no mortality from PPH in any of the groups. In view of this limitation, it is not acceptable to assess the association between PPH and BBA as significant. Further limitations in the study relate to the lack of accurate timing of delivery and antenatal data in the BBA group. A checklist was developed as a quality control intervention to improve the accuracy of patient information.

Apgar scores were not assessed in the BBA group due to the later arrival to the hospital; however, BBA mothers were asked if their babies cried or not after birth (Table 2). The study showed that more inborns had a lower apgar score compared to BBAs. This finding is limited as the concept of a woman's verbal report immediately after a traumatic experience may be arguably biased. The timing between birth and the actual cry was not explored. It was assumed that if she said her baby cried, it was documented as good a “apgar” score. This finding is therefore limited as it lacks the objective assessment of a trained professional providing the postdelivery neonatal score.

### 4.2. Recommendations

Health initiatives should focus on expanding coverage and care for pregnant women. Patient education and counselling on early antenatal booking remain the mainstay to reduce adverse outcomes associated with BBA. Early neonatal resuscitation is a simple but important intervention that can improve perinatal outcomes. Treatment and correction of maternal anaemia is an important component of antenatal care and can reduce prematurity and low birth weight. An efficient transport system needs to be implemented which will allow timeous attendance and presentation to the hospital and will reduce the BBA rate by improving accessibility to health care.

## 5. Conclusion

BBAs were associated with prematurity, low birth weight, hypothermia, and hypoglycaemia, which collectively contributed to significant neonatal morbidity. They were also associated with longer hospital stays than babies who were born in hospital. Mothers were also noted to be multiparous, unbooked, and from poor socioeconomic backgrounds.

## Figures and Tables

**Table 1:** Maternal characteristics.

Maternal characteristics	BBAs	Inborns	*p* value
*Parity*	*n* = 142	*n* = 127	≤0.001
Primigravida	37	75	
Multigravida	105	52	

*HIV status*	*n* = 142	*n* = 128	0.091
Positive	70	50	
Negative	72	78	

*ANC*	*n* = 142	*n* = 126	≤0.001
Booked	94	123	
Unbooked	48	3	

*Maternal anaemia*	*n* = 133	*n* = 111	≤0.001
Yes	59	41	
No	74	70	

BBAs: babies born before arrival; ANC: antenatal clinic.

**Table 2:** Characteristics of babies.

Baby's characteristics	BBAs	Inborns	*p* value
*Gender*	*n* = 129	*n* = 127	0.234
Male	53	62	
Female	75	65	

*Weight*	*n* = 142	*n* = 128	≤0.001
ELBW	3	2	
VLBW	9	0	
LBW	30	13	
NBW	99	112	
Large BW	1	1	

*Gestational age*	*n* = 135	*n* = 128	≤0.001
Preterm <37	73	23	
Term >37	62	105	

*Hypothermia*	*n* = 142	*n* = 128	0.018
Yes	13	3	
No	129	125	

*Hypoglycaemia*	*n* = 142	*n* = 128	0.029
Yes	12	3	
No	130	125	

*Apgar score/cry*	*n* = 142	*n* = 127	≤0.001
Poor	6	11	
Good	136	117	

BBAs: babies born before arrival; ELBW: extreme low birth weight; VLBW: very low birth weight; LBW: low birth weight; NBW: normal birth weight; Large BW: large birth weight.

**Table 3:** Perinatal outcomes.

Perinatal outcome	BBAs	Inborns	*p* value
Prematurity	73	23	≤0.001
Congenital abnormalities	1	1	0.941
Sepsis	2	3	0.670
Birth asphyxia	4	2	0.687
NICU admission	28	18	0.217
Birth trauma	5	1	0.217

*Length of hospital stay*			
Less than 24 hours	44	81	≤0.001
1–7 days	79	41	≤0.001
More than 7 days	17	6	≤0.001
Mortality	4	4	0.753

BBAs: babies born before arrival; NICU: neonatal intensive care unit.

**Table 4:** Maternal outcomes.

Maternal outcome	BBAs	Inborns	*p* value
Postpartum haemorrhage	110	0	≤0.001^*∗*^
1^st^ and 2^nd^ degree tears	40	25	≤0.001
3^rd^ degree tear	1	0	1.000
Retained placenta	2	0	0.499

BBAs: babies born before arrival. ^*∗*^See discussion on limitation of this finding.

## Data Availability

The datasets generated during and/or analysed during the current study are available from the corresponding author on reasonable request. All data generated or analysed during this study are included in this published article.

## Additional Points

BBAs had significant neonatal morbidity and were associated with prematurity, low birth weight, hypothermia, hypoglycaemia, and longer hospital stays.
